# Supplementary material for: Validity of Spatio-Temporal Gait Parameters in Healthy Young Adults Using a Motion-Sensor-Based Gait Analysis System (ORPHE ANALYTICS) during Walking and Running
Source: Sensors (Basel). 2022 Dec 28;23(1):331. doi: 10.3390/s23010331 (PMC9823871; doi:10.3390/s23010331)
Supplement: Supplementary file 1 [file sensors-23-00331-s001.zip › sensors-2025484-supplementary.pdf]

Supplementary Table S1. Status of trial execution and data acquisition

ID	sex	Walking			Running				
		2km/h	4km/h	6km/h	6km/h	9km/h	12km/h	12km/h forefoot strike	12km/h rearfoot strike
1	Male	●	●	●	●	●	●	●	●
2	Female	●	●	●	●	●			
3	Female	†	●	●	●	●	●		
4	Female	●	●	●	●	●	●	●	●
5	Male	●	●	●	●	●	●	●	●
6	Male	●	●	●	●	●	●	●	●
7	Male	●	●	●	●	●	●	●	●
8	Male	●	●	●	●	●	●	†	●
9	Male	●	*	*	*	*	*	*	*

●: Data with no deficits, †: Deficient left plantar-embedded sensor data, *: Deficient right plantar-embedded sensor data, no marker: the trial was not executed

Supplementary Table S2. Number of gait cycles from motion sensor data for the analysis

	ID	Walking						Running									
		2 km/h		4 km/h		6 km/h		6 km/h		9 km/h		12 km/h		12 km/h forefoot strike		12 km/h rearfoot strike	
		L	R	L	R	L	R	L	R	L	R	L	R	L	R	L	R
Plantar-embedded	1	29	29	36	37	43	43	55	56	54	58	53	58	68	67	67	67
	2	18	19	33	33	47	47	58	58	58	56	0	0	0	0	0	0
	3	0	17	31	32	31	32	55	55	58	59	54	53	0	0	0	0
	4	13	14	30	29	48	48	59	59	64	63	56	69	58	69	39	47
	5	13	12	27	27	39	38	40	39	44	44	47	47	53	53	45	46
	6	17	15	28	27	36	35	54	54	58	58	56	57	53	52	54	62
	7	14	15	27	27	35	35	52	52	52	53	60	59	57	57	64	65
	8	12	14	32	32	40	40	59	61	47	50	47	49	0	50	47	48
	9	12	13	23	0	34	0	43	0	41	0	44	0	44	0	45	0
Instep-mounted	1	29	29	36	37	43	42	55	55	58	58	57	58	68	66	67	66
	2	20	19	32	33	47	47	58	59	58	56	0	0	0	0	0	0
	3	14	17	31	32	31	30	56	55	57	58	53	53	0	0	0	0
	4	12	14	29	29	48	48	59	59	64	63	69	67	68	70	48	48
	5	13	12	27	27	39	39	40	40	44	44	47	47	53	53	45	45
	6	17	13	28	28	36	35	54	54	57	58	57	57	53	53	61	58
	7	14	15	27	27	35	35	52	52	53	53	60	60	57	50	64	65
	8	12	14	32	32	40	39	59	61	49	51	47	50	46	50	47	48
	9	13	13	23	23	34	34	43	43	41	41	44	44	41	44	45	46

L: left-side, R: right-side

Supplementary Table S3. Gait-cycle detection ratio for the conditions

Condition	Plantar-embedded		Instep-mounted	
Overall	99.16 %	(5981/5931)	99.54 %	(6343/6372)
Walking	99.94 %	(1675/1676)	99.83 %	(1758/1761)
Running	98.88 %	(4269/4303)	99.84 %	(4595/4611)
Forefoot strike	98.67 %	(740/750)	98.13 %	(841/857)
Rearfoot strike	99.16 %	(761/779)	99.28 %	(823/829)

Gait-cycle detection ratio (%) (number of gait cycles detected based on motion sensor data divided by the number of gait cycles detected based on motion capture data). Running: All running tasks, including the forefoot and rearfoot strike conditions

Supplementary Table S4. Percentage of gait cycles including outliers for the conditions

Condition	Plantar-embedded		Instep-mounted	
Overall	10.84 %	(578/5334)	9.52 %	(543/5701)
Walking	8.50 %	(122/1436)	9.25 %	(139/1503)
Running	11.70 %	(456/3898)	9.62 %	(404/4198)
Forefoot strike	13.36 %	(91/681)	13.60 %	(105/772)
Rearfoot strike	11.06 %	(77/696)	6.64 %	(50/753)

Percentage of gait cycles including outliers: number of gait cycles excluded in outlier processing divided by the number of gait cycles detected by the motion sensor. Running: All running tasks, including the forefoot and rearfoot strike conditions

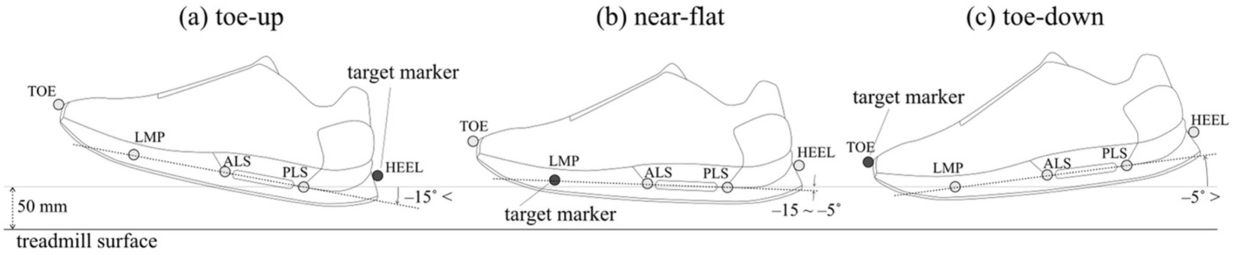

Supplementary Figure S1. Definition of target markers for detecting the initial foot contact timing. (a) The HEEL marker was assigned as the target marker when the angle between the anteroposterior axis of the motion sensor and global horizontal plane was less than –15° (toe-up). (b) The LMP marker was assigned when the angle ranged from −15° to −5° (near-flat). (c) The TOE marker was assigned when the angle was more than –5° (toe-down).

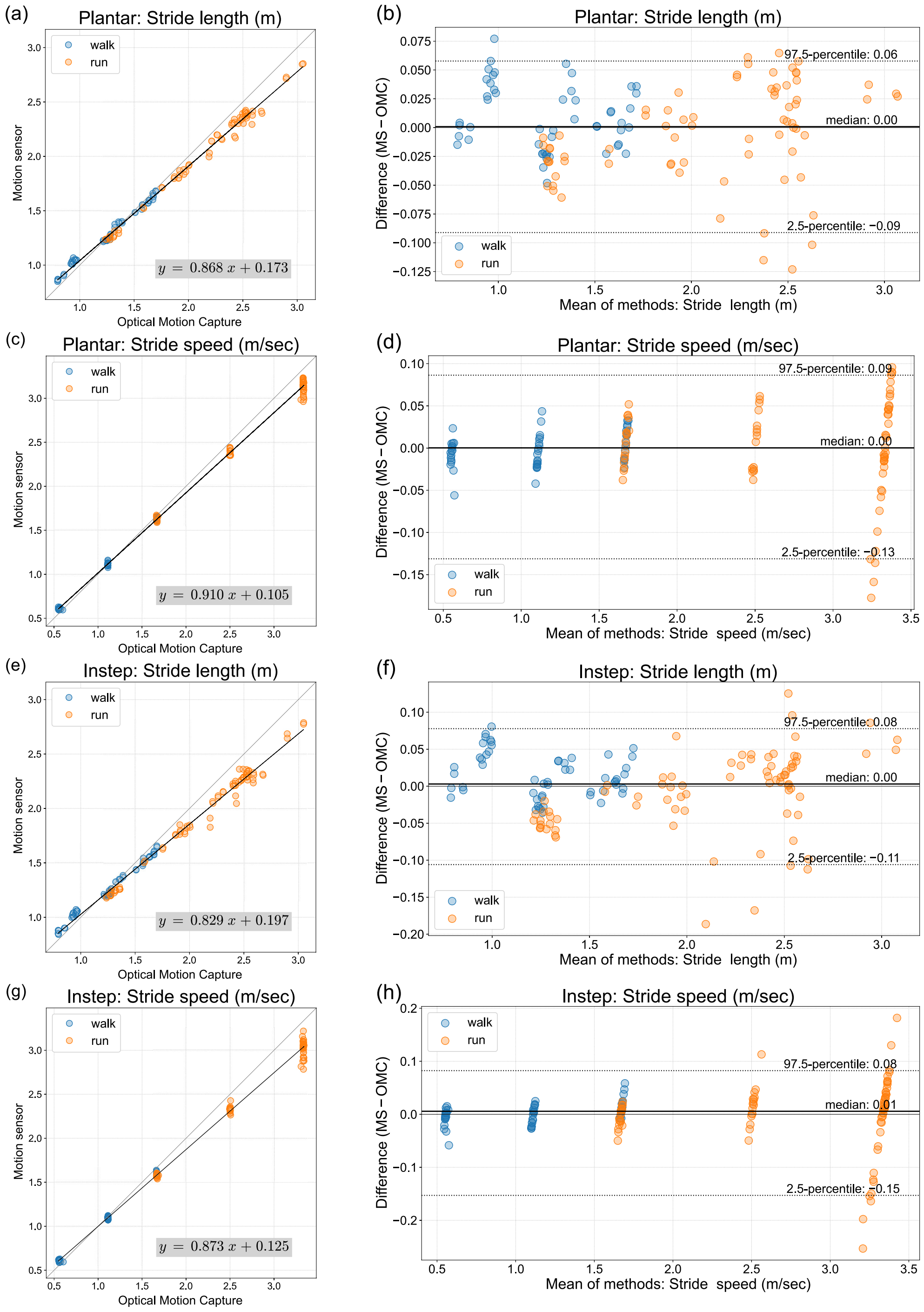

Supplementary Figure S2. Scatter plots and regression lines of the stride length and speed from ORPHE ANALYTICS and the optical motion capture and the Bland–Altman plots of corrected data using regression lines. (a) Scatter plot: stride length (plantar); (b) Bland–Altman plot: stride length (plantar); (c) scatter plot: stride speed

(plantar); (d) Bland–Altman plot: stride speed (plantar); (e) scatter plot: stride length (instep); (f) Bland–Altman plot: stride length (instep); (g) scatter plot: stride length (instep); (h) Bland–Altman plot: stride length (instep). MS: motion sensor, OMC: optical motion capture. The IQR% of stride length (plantar), stride speed (plantar), stride length (instep), and stride speed (instep) were 3.2, 2.6, 2.0, and 3.6, respectively.
